# The Relationship Between Perceived Stress and Subjective Cognitive Decline During the COVID-19 Epidemic

**DOI:** 10.3389/fpsyg.2021.647971

**Published:** 2021-08-05

**Authors:** Anja Podlesek, Luka Komidar, Voyko Kavcic

**Affiliations:** ^1^Department of Psychology, Faculty of Arts, University of Ljubljana, Ljubljana, Slovenia; ^2^Institute of Gerontology, Wayne State University, Detroit, MI, United States; ^3^International Institute of Applied Gerontology, Ljubljana, Slovenia

**Keywords:** COVID-19, SARS-CoV-2, stress, emotions, cognitive complaints, physical health, cognitively normal adults

## Abstract

During the outbreak of the COVID-19 epidemic, fear of disease and its consequences, recommended lifestyle changes, and severe restrictions set by governments acted as stressors and affected people’s mood, emotions, mental health, and wellbeing. Many studies conducted during this crisis focused on affective and physiological responses to stress, but few studies examined how the crisis affected cognition. The present cross-sectional study examined the relationship between physiological, affective, and cognitive responses to the epidemic. In an online survey conducted at the height of the first wave of the epidemic in Slovenia (April 15–25, 2020), 830 Slovenian residents aged 18–85 years reported the effects of stressors (confinement, problems at home, problems at work, lack of necessities, and increased workload), experienced emotions, generalized anxiety, perceived stress, changes in health, fatigue and sleep quality, and perceived changes in cognition during the epidemic. Risk factors for stress (neuroticism, vulnerability, general health, gender, and age) were also recorded. We hypothesized that stressors and stress risk factors will be related to subjective cognitive decline, with negative emotions, generalized anxiety, perceived stress, and physical symptoms acting as mediator variables. On average, the results showed a mild subjective cognitive decline during the epidemic. In structural equation modeling, 34% of its variance was predicted by the mediator variables, with negative emotions and physical symptoms having the largest contribution. Stress risk factors were predictably related to the four mediator variables. Among the stressors, confinement showed the strongest effect on the four mediator variables, implying the importance of thoughtful communication about necessary restrictive measures during emergency circumstances. The results of this study indicate that the possibility of altered cognitive function should be considered when planning work and study activities during the epidemic.

## Introduction

On March 11, 2020, the World Health Organization declared a COVID-19 pandemic (Ghebreyesus, 2020). Slovenia declared the epidemic on March 12, 2020 (Government of the Republic of Slovenia, 2020a). Due to the sudden nature of the COVID-19 outbreak and high infectiousness of the SARS-CoV-2 virus, the Slovenian Government implemented several restrictions. In addition to the introduction of the minimum physical distance of 1.5 m and the mandatory use of face masks, freedom of movement was restricted to small municipalities, people were expected to self-isolate in their homes, most economic activities were stopped, and public life was closed. Public services such as public transport and health services were restricted. Shops were closed, except for grocery stores. Educational institutions were closed and switched to online teaching. According to Eurofound (2020), in April and May 2020, 10% of people reported that they worked from home every day or several times a week before the epidemic, and 23% reported that they started working from home as a result of the situation, with 17% of residents reporting that their working hours increased and 46% reporting that they decreased. The media covered the crisis extensively, and the amount of information about COVID-19 and the ever-changing measures to limit the outbreak was overwhelming. In retrospect, it could be assumed that Slovenians quickly adapted to the emergency measures and followed strict restrictions and recommended lifestyle changes, making Slovenia the first European country to announce the end of the first wave of the epidemic on May 15, 2020 (Government of the Republic of Slovenia, 2020b). However, the implemented restrictions affected people’s behavior and psychological wellbeing (Lep and Hacin Beyazoglu, 2020).

### Stress During the COVID-19 Epidemic

The course of the COVID-19 pandemic as a global, prolonged health crisis was unpredictable and beyond control of individuals. It caused stress to many people, and the measures taken to restrict the spread of the virus had many negative effects. A plethora of studies conducted around the world, including in Slovenia (Lep and Hacin Beyazoglu, 2020), showed that the COVID-19 crisis affected people’s mood and emotions (Xiao et al., 2020), leading to decreased subjective wellbeing (Möhring et al., 2020; Ammar et al., 2021b; Paredes et al., 2021), sleep disturbances (Gualano et al., 2020; Pinto et al., 2020; Saraswathi et al., 2020; Ammar et al., 2021b), and increased prevalence of psychiatric conditions, such as generalized anxiety disorder, depression, or post-traumatic stress symptoms (Fu et al., 2020; Liu et al., 2020). The confinement during the epidemic also resulted in an increase of social isolation, physical inactivity (the hours of daily-sitting) and unhealthy diet behaviors (Ammar et al., 2021b). However, only few studies examined how people’s cognition changed during the COVID-19 crisis. Batty et al. (2020), in a prospective cohort study using UK Biobank data, found that a higher risk of hospitalization for COVID-19 was observed for participants with lower performance on two tests of cognitive function – verbal and numerical reasoning and reaction speed – which the authors suggested could be potential markers of health literacy. Commenting on the consequences of the COVID-19 pandemic, Boals and Banks (2020, p. S255) suggested that increases in stress and anxiety are likely to impair cognitive functioning. They wrote that “anecdotally, in the time since the pandemic, students and colleagues have shared that they have had trouble staying focused and productive.” Our own observation was consistent with this comment – many people in our setting who worked from home reported being more tired and having more trouble multitasking. In the present study, therefore, we wanted to investigate how different stressors, i.e., different aspects of the COVID-19 crisis, such as stress related to changes in living and working conditions, affected the adult population of Slovenia. We wanted to examine the relationship between different types of stress responses, including physiological, affective, and cognitive responses. More specifically, we were interested in subjectively reported change in cognitive performance.

### Physiological, Affective and Cognitive Responses to Stress

Stress affects mood and emotions, cognition, behavior, wellbeing, and health (Schneiderman et al., 2005). It activates the hypothalamic–pituitary–adrenal (HPA) axis, resulting in physiological responses such as increased cortisol secretion (Kemeny, 2003), heart rate, respiratory rate, blood pressure and muscle tension, making an organism ready for action. Affective responses to stress include negative affect (e.g., feelings of tension, panic, feeling overwhelmed, irritability, restlessness, anger, guilt, sadness, grief, and depression) or positive affect (e.g., feelings of happiness, enthusiasm, contentment, and excitement; Zhaoyang et al., 2020). Cognitive responses to stress include mental slowing, confusion, narrowing of focus, difficulty concentrating, memory impairment (forgetfulness), increased or decreased awareness of one’s surroundings, general negative thinking, intrusive and repetitive thoughts and images, constant worry, difficulty making decisions, poor abstract thinking, disturbed thinking, difficulty identifying familiar objects or people, loss of orientation in time and place, and changes in learning and memory (Becker et al., 1973; Bryce, 2001; Kemeny, 2003).

The direct effect of stress on cognitive functions is not entirely clear, and research examining the effects of stress on executive functions has yielded counterintuitive results (see Shields et al., 2016). Studies found that acute and chronic psychological stress can induce structural and functional changes in the adult brain and impair memory and executive functions (Diamond, 2013; Chattarji et al., 2015; Shields et al., 2016). Executive functions encompass the higher cognitive processes that enable cognitive control, i.e., planning, thinking ahead, and goal-directed action, and include working memory (the ability to retain information in memory and update it regularly), inhibition (the ability to inhibit thoughts or prepotent responses in order to selectively attend to task-relevant information and engage in goal-directed behavior), and cognitive flexibility (the ability to flexibly switch between cognitive rules or ways of thinking; Miyake et al., 2000; Diamond, 2013). In a meta-analysis on the effects of acute stress on executive functions, Shields et al. (2016) showed that stress impairs working memory and cognitive flexibility and that these effects are moderated by sex; stress was also found to impair cognitive inhibition but increase response inhibition, suggesting that stress contributes to a cognitive state of automatic, reactive processing and more alert executive motor control that allows a person to quickly engage with or escape from the current stressor. Qin et al. (2009)found that experimentally induced acute stress leads to deficits in working memory, increased catecholamine and cortisol levels, reduced activation of the dorsolateral prefrontal cortex, and a reallocation of neural resources away from executive function networks. Liston et al. (2009) found in undergraduates that a month-long psychosocial stress related to exams impaired attentional shifting and disrupted functional connectivity within a frontoparietal network mediating attentional shifting, although these impairments were reversible after the stress ended. In general, stress causes a focus on the here and now, resulting in impaired retrospective and prospective memory (Bourne and Yaroush, 2003). The time span from which knowledge can be easily retrieved and used in a given context shrinks as stress levels increase (Bourne and Yaroush, 2003). Functioning during stress may be adaptive in the short term, biasing processing in favor of a single salient stimulus (Liston et al., 2009) or the current stressor to allow an organism to effectively cope with the current unstable circumstances (Shields et al., 2016).

### The Interconnectedness of Different Types of Responses to Stress

Emotion and cognition are deeply intertwined (Okon-Singer et al., 2015). Intense negative emotions can interfere with focusing on, encoding, and retrieving important information. Emotions that cause high arousal can divert attention to specific stimuli and affect attentional focus, working memory, learning, reasoning, problem solving, and cognitive control, especially inhibition (Harlé et al., 2013; Okon-Singer et al., 2015). Individuals with emotional disorders, such as anxiety and depression, show impaired cognitive processes (Mathews and MacLeod, 2005). Anxiety can distort attentional processing, because it narrows the range of perceived stimuli and focuses attention on the threat. It biases evaluations of stimulus valence (Yiend, 2010), as well as individual perceptions of reality (Spielberger, 1966). Conversely, cognition can also alter, activate, and inhibit emotions; effortful cognitive strategies, such as reappraising the situation in a more positive light, can be used to cope with and regulate negative emotions (Kryla-Lighthall and Mather, 2009; Cole et al., 2014; Okon-Singer et al., 2015; Tyng et al., 2017).

Complex relationships exist between stress, fatigue, sleep, self-perceived health status, and performance (De Vries et al., 2003; Taylor and Dorn, 2006; Kocalevent et al., 2011; Khanade and Sasangohar, 2017). Sleep and stress interact in a bidirectional manner. Stress causes changes in metabolism through activation of the HPA axis and increased release of glucocorticosteroids, leading to impaired sleep (Van Reeth et al., 2000). This in turn affects the regulation of HPA axis activity, which indirectly modulates arousal (Hirotsu et al., 2015). Sleep disturbances affect how we respond to emotional events during the day, and conversely, responses to past emotional events affect sleep quality (Altena et al., 2016). Stress, anxiety, and depression are associated with fatigue and poorer subjective sleep quality (Van Reeth et al., 2000; Valerio et al., 2016; Thorsteinsson et al., 2019; Cox and Olatunji, 2020; Xiao et al., 2020). Although some studies found no association between subjective sleep quality and cognitive performance (Zavecz et al., 2020), many studies report that sleep quality also affects cognition. It is associated with problems in attention, working memory, and executive functions (Scullin and Bliwise, 2015). Sleep loss and deprivation have been found to impair performance on cognitive tasks involving vigilance and attention, working and long-term memory, learning, logical reasoning, arithmetic calculations, pattern recognition, complex verbal processing, and decision making (Krueger, 1989; Alhola and Polo-Kantola, 2007). Partial sleep restriction deteriorates memory encoding and the ability to learn declarative information (Cousins et al., 2018). Following sleep deprivation, cognitive impairments are thought to be mediated through decreased alertness, attentional lapses, and slowed responses (Alhola and Polo-Kantola, 2007). Thus, cognition can be impaired by stress, fatigue, and decreased sleep quality.

### Individual Differences in Stress Reactivity

Responses to the same stressor are not the same for all individuals. Stress occurs when individuals perceive that environmental demands tax or exceed their adaptive capacity (Cohen et al., 1983, 1997), so stressful experiences can be viewed as person-environment transactions, the outcome of which depends on both the stressor and the individual (Kemeny, 2003). In terms of the nature of stressors, circumstances that are perceived as uncontrollable, ambiguous, novel, and durable are more likely to activate a stress response (Kemeny, 2003; Dickerson and Kemeny, 2004). The influence of the external stimulus is mediated by the characteristics of the individual, such as primary appraisal of the stimulus as a threat vs. challenge (Kemeny, 2003), lack of confidence (Farrer et al., 2016), coping mechanisms, self-esteem and social skills (Uchino, 2009), the efficacy of coping efforts (Schneiderman et al., 2005; Pallavicini et al., 2013), social support (Cohen et al., 2000; Cohen, 2004; Qi et al., 2020) and social capital (Xiao et al., 2020), appraisal of psychosocial resources to cope with the stressor, e.g., appraisal of coping skills, personality factors, intellectual resources, financial resources, environmental resources (Kemeny, 2003), and perceived control over potentially negative events (Gallagher et al., 2014). Stress responses, including threat appraisals, negative and positive affect, and task performance, are also related to personality traits, such as neuroticism (Schneider, 2004), extraversion, and openness (Schneider et al., 2012). In addition, larger stress responses are associated with low socioeconomic status, female gender, younger age (Scott et al., 2013; Novais et al., 2017), and poorer physical wellbeing prior to the onset of the stressor (Kocalevent et al., 2011).

### Subjective Cognitive Decline

Subjective cognitive complaints are everyday memory and related cognitive concerns expressed by people with or without objective evidence of cognitive impairment and are common across all age groups (Jacob et al., 2019). Subjective cognitive decline is not only predictive of Alzheimer’s disease dementia (Jessen, 2014), but is also associated with numerous other conditions, including normal aging (dos Santos et al., 2012), depression and anxiety (Hill et al., 2016), pregnancy (Crawley et al., 2008), substance use and medication (Jessen et al., 2014), and physical illness (Jacob et al., 2019). In older individuals, memory complaints without actual cognitive decline have been found to be associated with physical health problems, depressive and anxiety symptoms, higher perceived stress and lower mastery (control of potential problems in life), ineffective coping, and high neuroticism (Comijs et al., 2002; Steinberg et al., 2013). Stenfors et al. (2013) found that subjective cognitive complaints in healthy, working non-elderly adults were related to emotional exhaustion, burnout, mental fatigue, disturbed sleep, awakening problems, depressive symptoms, and poorer executive cognitive functioning. Jacob et al. (2019) found in a large nationally representative survey that subjective concentration and memory complaints were predicted by the number of stressful life events, perceived stress, depression, anxiety disorders, sleep problems, and physical health problems (multiple chronic diseases).

### The Aim of This Study

Previous studies have rarely examined the effects of chronic stress on human cognitive functions because it would be difficult and unethical to experimentally manipulate such stress conditions (Shields et al., 2016). Thus, most of the evidence on the effects of stress and emotion on cognition has been obtained in short-term experimental studies, in clinical populations, or in selected samples with long-term exposure to stress, such as certain work groups (nurses and shift workers). Less is known about how prolonged collective situational uncertainty, such as that experienced by society at the time of the COVID-19 epidemic, can affect cognitive functioning and its perception. We therefore aimed to investigate whether subjective cognitive complaints during the crisis can be predicted by the physiological and affective responses to stress.

**Figure 1:**
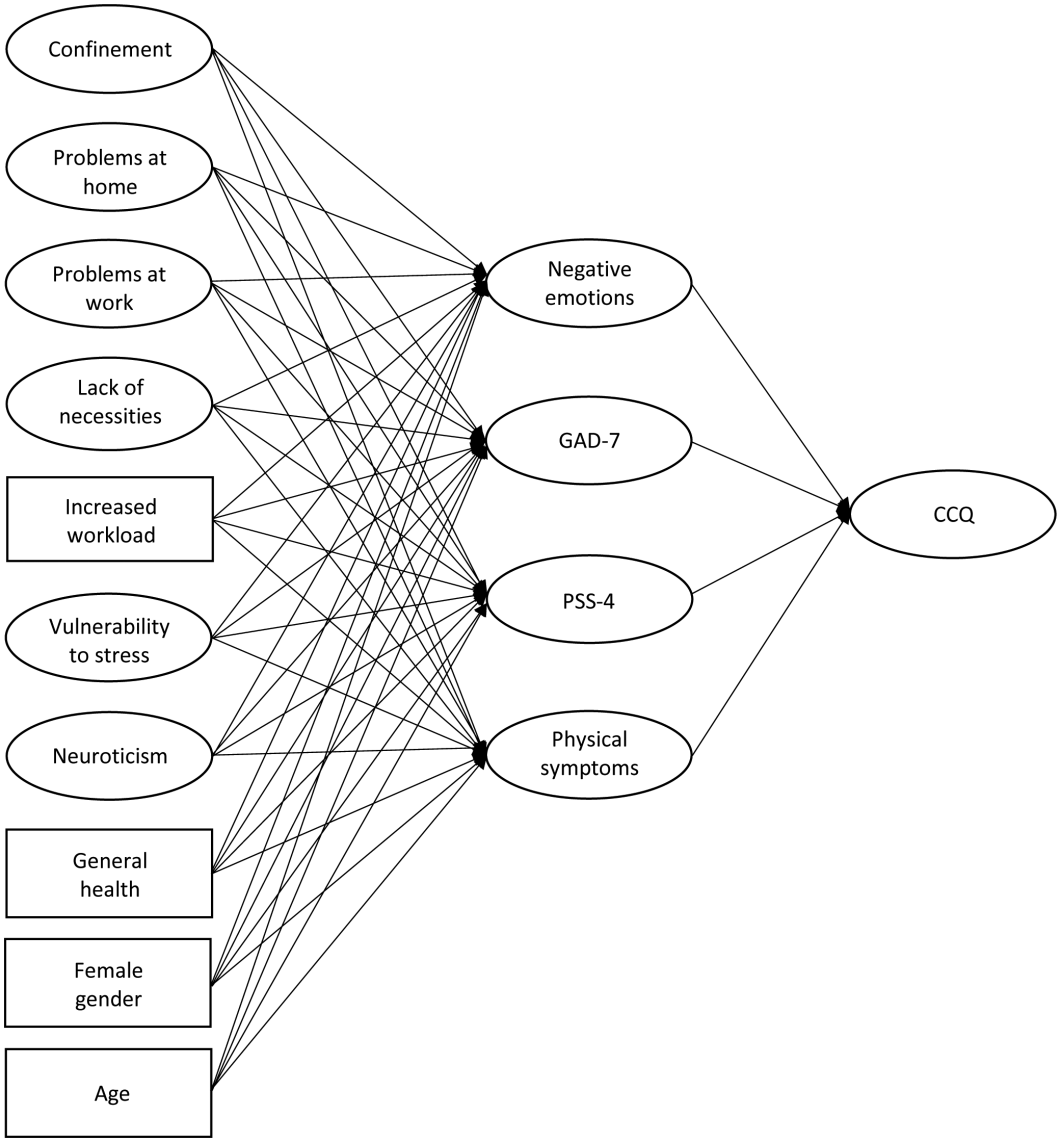
A simplified representation of the structural model for predicting subjective cognitive change. Negative emotions, generalized anxiety (GAD-7), perceived stress (loss of perceived control; PSS-4), and physical symptoms mediate the effects of stressors and stress risk factors on subjective cognitive change (CCQ).

Based on the literature presented, we developed a model of subjective cognitive change during the COVID-19 epidemic, as shown in Figure 1. We expected that the perceived impact of stressors caused by the COVID-19 epidemic would be related to more intense physiological responses leading to physical symptoms (including fatigue, sleep disturbance, physical pain, and worsening of illness) and affective responses (including negative emotions, generalized anxiety, and perceived stress), which in turn would be related to higher levels of subjective cognitive decline associated with impaired attention, memory, and cognitive control. We also expected stress risk factors, such as neuroticism, vulnerability to stress, poorer general health, female gender, and younger age to contribute positively to physiological and affective responses to stress. In addition, we were interested in the association between subjective cognitive change and various demographic characteristics.

## Materials and Methods

### Participants

To assess perceived stress and responses to stress during the COVID-19 epidemic, we designed an online survey. The survey was open on the Slovenian online survey platform 1KA (2020) for 10 days, from April 15 to 25, 2020, at the peak of the first wave of the epidemic, when the trend of newly detected COVID-19 cases began to level off. It was accessed by 1,290 individuals. Of 1,135 who began filling it out, 1,072 (94%) completed at least part of the survey, 881 (77%) responded to the final section of the survey, and 830 participants had complete data on the variables included in the analyses. Table 1 shows a description of the final sample by gender, age, education, marital status, and employment status. In the general population of Slovenia, the share of the same age categories as in our study was 8, 15, 18, 17, 17, 13, and 11% in the first half of 2020 (Statistical Office of the Republic of Slovenia, 2020). In our sample, the oldest age category was underrepresented, which is most likely related to the online administration of the survey.

**Table 1:** Description of nominal variables and comparison of subgroups on the Cognitive Change Questionnaire (CCQ) score.

Variable	*n* (%)	*M* (*SD*)	Result of the statistical test	Effect size	1 − *β*
Gender			*t*(407.41) = −1.52, *p* = 0.129	*d* = −0.09	0.34
Male	183 (22)	4.14 (0.46)			
Female	647 (78)	4.19 (0.62)			
Age (years)			*F*(6, 823) = 0.83, *p* = 0.549, *MSE* = 0.35	*η*^2^ = 0.01	0.88
18–24	86 (10)	4.25 (0.65)			
25–34	162 (20)	4.12 (0.58)			
35–44	167 (20)	4.21 (0.68)			
45–54	147 (18)	4.16 (0.65)			
55–64	123 (15)	4.14 (0.60)			
65–74	128 (15)	4.23 (0.35)			
75 and more	17 (2)	4.18 (0.25)			
Education			*t*(238.09) = −0.18, *p* = 0.856	*d* = −0.02	0.86
High school or less	160 (19)	4.18 (0.60)			
More than high school	669 (81)	4.18 (0.59)			
Relationship status			*t*(378.26) = 0.69, *p* = 0.489	*d* = 0.06	0.59
In a relationship^1^	592 (71)	4.19 (0.56)			
Not in a relationship^2^	236 (28)	4.16 (0.66)			
Employment status			*F*(4, 788) = 1.94, *p* = 0.101, *MSE* = 0.34	*η*^2^ = 0.01	0.66
Student	105 (13)	4.21 (0.61)			
Working regularly/from home	354 (45)	4.15 (0.61)			
Working less than before the epidemic	96 (12)	4.07 (0.68)			
Not working^3^	68 (9)	4.22 (0.61)			
Retired	170 (21)	4.25 (0.43)			
General health			*F*(3, 826) = 3.82, *p* = 0.010, *MSE* = 0.35	*η*^2^ = 0.01	0.43
Poor	9 (1)	4.20 (0.39)			
Fair	165 (20)	4.31 (0.60)			
Good	411 (50)	4.17 (0.59)			
Excellent	245 (30)	4.12 (0.59)			
Increased workload			*t*(329.63) = 1.36, *p* = 0.174	*d* = 0.11	0.53
Yes	207 (25)	4.23 (0.63)			
No	623 (75)	4.17 (0.58)			

1 *Married or in a relationship*.

2 *Single, divorced, separated, or widowed*.

3 *Unemployed, on a sick leave, on a maternity leave, or on a furlough*.

### Instruments

This research was planned and conducted in an international group of researchers from China, the United States, and Slovenia, who sought to gain insight into a wide range of experiences with the COVID-19 outbreak among participants from different countries who faced different measures to prevent the spread of the SARS-CoV-2 virus. The common core of the survey was negotiated and took into account the different contexts in the participating countries. It included a combination of self-constructed questions and questions from previously validated questionnaires. The aim was to cover many different aspects of the experiences while being manageable and time efficient for participants. In this paper, we report only the results of the study conducted in Slovenia on selected variables related to our defined research problem.

#### Perception of Stressors

Participants rated on a 5-point scale how much they experienced various stressors or difficulties that negatively affected their mood or emotions during the COVID-19 pandemic (1 – not at all, 2 – a little, 3 – a moderate amount, 4 – a lot, and 5 – a great deal; ‘Not applicable’ (N/A) was also added but later changed to 1 because if a particular factor was not relevant to the participants, it did not affect them). The first type of stressor related to the specifics of the epidemic crisis and lockdown: time spent indoors, media coverage, and restricted movement. The second type of stressor was related to difficulties at home: family relationships, intimate partner relationship, and reduced privacy. The third type of stressor included work-related problems, academic problems, and economic problems. The fourth type of stressor was lack of necessities: personal protective equipment, food, medicine, and access to a doctor. Participants also reported whether their workload had increased during the epidemic (Yes/No).

#### Physical Symptoms

To assess participants’ physiological reactions to stress, we asked them to rate on a 5-point scale (1 – not at all, 2 – a little, 3 – a moderate amount, 4 – a lot, and 5 – a great deal; N/A was changed to 1) how much they experienced physical pain, worsening of illness, and fatigue/sleepiness during the epidemic. They also rated how tired they were and how well they slept during the COVID-19 outbreak compared to before the outbreak. These changes in fatigue and sleep quality were rated on 5-point scales (1 – much less/better, 2 – less/better, 3 – same, 4 – more/worse, and 5 – much more/worse).

#### Affective Responses to Stress

To assess participants’ affective state and stress during the COVID-19 epidemic, we used the GAD-7 scale and self-constructed inventories of emotional states and vulnerability indicators. A Brief Measure for Assessing Generalized Anxiety Disorder – GAD-7 (Spitzer et al., 2006) – is a clinical screening self-report measure consisting of 7 items. Participants rated on a 4-point scale how often they experienced the listed symptoms since the beginning of the epidemic (0 – never, 1 – several days, 2 – over half the days, and 3 – nearly every day). When used for clinical assessment, item responses are summed. In the study by Spitzer et al. (2006), the instrument demonstrated good reliability (Cronbach’s alpha coefficient was 0.92 and test–retest intraclass correlation was 0.83), construct validity (a higher score was strongly associated with multiple domains of functional impairment and disability days), and factorial validity (the scale differentiated symptoms of generalized anxiety from those of depression).

In addition to GAD-7, participants rated how much they experienced the following emotions on a 5-point response scale (1 – not at all, 2 – a little, 3 – a moderate amount, 4 – a lot, and 5 – a great deal): Anger at others, anger at self, sadness, fear, worry, annoyance, depression, distracted thinking, longing for normality, and loneliness.

Participants also completed the Perceived Stress Scale – PSS-4 (Warttig et al., 2013). PSS-4 is a 4-item version of a self-report questionnaire developed by Cohen et al. (1983) to measure how often in the past month the person felt nonspecific appraised stress and was unable to control the important things in their life (1 – never, 2 – almost never, 3 – sometimes, 4 – fairly often, and 5 – very often). The higher the score on the PSS-4, the more the respondents perceive that the demands exceed their ability to cope (Warttig et al., 2013). We therefore considered the responses on this scale to be most indicative of the loss of perceived control during the crisis. Cohen et al. (1983) found high correlations of PSS scores with depressive symptomatology and stress measures in samples of college students. Warttig et al. (2013) found acceptable psychometric properties of the PSS-4 on an English sample (Cronbach’s alpha was 0.77) and low to moderate negative correlations with perceived health status, social support, and age, with women reporting higher stress than men.

#### Cognitive Responses to Stress

Because we were in a lockdown and faced with emergent and rapidly changing situations, we could not conduct objective cognitive tests. There were also no data available on residents’ cognitive function prior to the epidemic. Therefore, we opted for self-report of the changes people observed in their everyday cognitive function. We looked for questionnaires that were general enough and did not ask about instrumental daily activities, since we targeted healthy adults. We could not find a questionnaire that could be easily applied to the situation, so we decided to develop a new instrument. The survey was long and asked about many different variables, so we wanted to keep the instrument on cognitive changes short. To capture self-perceived changes in cognition, we drew on the Working Memory Questionnaire (Vallat-Azouvi et al., 2012) and the Teenage Executive Functioning Inventory – TEXI (Thorell et al., 2020), and compiled nine questions asking about speed of information processing, short-term storage, prospective memory, attention, and executive control (see Table 2). We had no information about participants’ previous cognitive functioning and were not interested in absolute levels of functioning, so we decided to ask participants directly about the changes they observed in their cognition during the COVID-19 epidemic. They were asked to compare their current state (the state during the epidemic) with the state before the epidemic, using a 7-point scale. For items 1–5, the following scale was used: 1 – much less often, 2 – less often, 3 – a little less often, 4 – same as before, 5 – a little more often, 6 – more often, and 7 – much more often (than before). For items 6–9, the following scale was used: 1 – much easier, 2 – easier, 3 – a little easier, 4 – same as before, 5 – a little harder, 6 – harder, and 7 – much harder (than before). We will refer to these questions as the Cognitive Change Questionnaire (CCQ).

**Table 2:** Item content, descriptive statistics, and standardized factor loadings from the one-factor measurement model of the CCQ.

S. No.	Item	Cognitive function	*M*	*SD*	Skew	Kurt	*λ*
1.	How quickly have you performed your usual activities?^†^	Processing speed	3.59	1.01	0.13	1.03	−0.44^***^
2.	How often have you had to re-read something to understand it?	Attention and working memory	4.13	0.74	−0.17	4.19	0.69^***^
3.	How often have you felt disturbed when something unexpectedly interrupted your activity?	Executive control (inhibition)	4.15	0.84	−0.25	3.32	0.74^***^
4.	How quickly do you get tired doing activities that require a lot of attention (e.g., reading, studying, following an instruction manual)?	Attention	4.23	0.92	0.12	2.03	0.76^***^
5.	How often have you forgotten things that should be done in the immediate future?	Prospective memory	4.11	0.87	−0.32	3.24	0.76^***^
6.	How much easier or harder have you found it to »multi-task«, that is, to focus your attention on several things at once (e.g., listening to the news *and* cooking from a recipe, working on the computer *and* talking to someone)?	Attention and executive control (shifting)	4.14	0.69	0.15	5.86	0.83^***^
7.	How much easier or harder has it been for you to plan future activities and organize things (e.g., scheduling appointments, sorting documents, paying bills, filling out forms)?	Executive control (planning and organization)	4.21	0.99	−0.19	1.58	0.60^***^
8.	How much easier or harder has it been for you to remember everything someone asks you to do?	Memory	4.11	0.70	0.39	5.65	0.84^***^
9.	How much easier or harder has it been for you to switch between tasks when doing several things at once?	Executive control (shifting)	4.15	0.73	0.45	4.08	0.83^***^

*N = 830. Min and max were 1 [much less (often)/easier than before] and 7 [much more (often)/harder than before], respectively, for all CCQ items. Skew, skewness; Kurt, kurtosis; λ, standardized factor loadings*.

† *This item was scored reverse before calculating the scale score*.

*** *p < 0.001*.

#### Stress Risk Factors

Participants’ vulnerability to stress was assessed by their ratings on a 5-point scale (1 – not at all; 5 – a great deal) of the extent to which their sense of imbalance, self-denial, lack of resilience, vulnerability, tendency to suppress emotions, lack of family support, lack of social support, perfectionistic tendencies, poor adaptability, lack of self-confidence, and lack of coping skills played a role in their negative emotions related to the COVID-19 epidemic. Their neuroticism was rated on the same scale based on their responses to questions how much it describes them as a person to be emotionally stable and worry free. They also rated their general health (not limited to the period of the epidemic) on a 4-point scale (1 – poor, 2 – fair, 3 – good, and 4 – excellent).

The survey also asked participants about their gender, age category, employment status, relationship status, and education level.

### Procedure

The Ethics Committee of the Faculty of Arts at University of Ljubljana approved the study (approval No. 184-2020). Snowball sampling was used to recruit participants. The researchers AP and VK sent invitations to their personal email contacts and posted announcements on several Facebook pages and on websites of various organizations. Participants were asked to forward the invitation to their relatives, friends, and acquaintances. Participation in the survey was voluntary. Participants received no benefits for participating in the study. They were introduced to the purpose of the survey and gave their consent to participate in the study by clicking a specific button on a survey webpage. To avoid loss of motivation and dropout from participation in the survey, answering most questions was not mandatory and could be skipped if desired. On average, participation in the survey took 15 min.

### Data Analysis

Frequency distributions for each item were examined and descriptive statistics were calculated.

Structural equation modeling was used to evaluate the following theoretical model (Figure 1): (i) Each of the four types of responses to stress (negative emotions, generalized anxiety, perceived stress or loss of perceived control, and physical symptoms) was predicted by the perceived impact of external stressors that occurred during the epidemic (confinement, problems at home, problems at work, lack of necessities, and increased workload); (ii) each of the four types of responses to stress was also predicted by stress risk factors: vulnerability to stress, neuroticism, general health, gender, and age (age category means were analyzed); and (iii) four types of responses to stress predicted subjective cognitive change during the COVID-19 epidemic. Perceptions of stressors and stress risk factors were thus entered as predictors (exogenous variables) in the structural equation model. Physical symptoms, negative emotions, generalized anxiety, and perceived stress were treated as endogenous variables and were also considered mediators between predictors and subjective cognitive change. All observed variables (variables listed in Supplementary Table 1, along with gender, age, general health, and increased workload) were entered into the model simultaneously. Due to the ordinal nature of the observed variables we used the robust weighted least squares estimator (WLSMV), implemented by the *cfa* function in the R *lavaan* package (Rosseel, 2012). The following cutoff values were considered indicative of acceptable fit of the model to the data (Marsh et al., 2004): CFI and TLI > 0.90; RMSEA and SRMR < 0.08.

The reliability of the scales was calculated using the *omega* function in the R *psych* package (Revelle, 2015). Because the measurement models supported the unidimensional structure of the latent constructs under study, we calculated Cronbach’s alpha coefficient as a measure of internal consistency. We also report the McDonald’s omega total, which is a better choice for reliability estimation in the presence of skewed item distributions and the absence of tau-equivalence, i.e., in the case of different factor loadings (Trizano-Hermosilla and Alvarado, 2016).

Responses to items measuring a specific construct were averaged and descriptive statistics were calculated for such scale scores. Welch’s *t* test and ANOVAs were used to compare the CCQ score in subsamples based on demographic variables (gender, age, education, relationship status, and employment status) and general health status.

All statistical hypotheses were tested at the significance level of 5%.

## Results

### The Fit of the Proposed Structural Equation Model

The model tested fit the observed data closely enough, *χ*^2^(1948) = 5653.79, *p* < 0.001, CFI = 0.906, TLI = 0.912, RMSEA = 0.048, 95% confidence interval for RMSEA = 0.046–0.049, *p*(RMSEA ≤ 0.05) = 0.991, and SRMR = 0.057.

Testing the measurement models of all included constructs confirmed their one-dimensional structure. Table 2 shows the estimated parameters in the measurement model related to the CCQ, and Supplementary Table 1 shows the estimated parameters in the measurement models for other latent constructs. For most items, factor loadings were high and consistent with expectations. In the CCQ, all items except item 1 loaded highly on the general factor. Excluding this item would not increase the reliability of the instrument, so we decided to keep it.

The scales measuring subjective cognitive change, negative emotions, generalized anxiety, and vulnerability to stress showed good reliability (Table 3), with Cronbach’s alpha coefficients of internal consistency exceeding the value of 0.88. The reliability of other scale scores was lower, but considering a small number of items on these scales, we concluded that their reliability was also acceptable (Table 3).

**Table 3:** Descriptive statistics and the reliability of the scale scores (*N* = 830).

Construct	Number of items	Scale	*M*	*SD*	Skew	Kurt	Reliability		
							*α*	*ω*	AVE
CCQ	9	1–7	4.18	0.59	−0.07	5.16	0.88	0.88	0.53
Negative emotions	10	1–5	2.27	0.70	0.51	−0.16	0.88	0.88	0.52
GAD-7	7	0–3	0.61	0.54	1.04	0.92	0.88	0.88	0.65
PSS-4	4	1–5	2.45	0.66	0.37	0.31	0.67	0.68	0.41
Vulnerability to stress	11	1–5	1.78	0.66	0.91	0.30	0.90	0.90	0.56
Neuroticism	2	1–5	2.94	0.77	0.04	−0.14	0.62	0.62	0.51
Confinement	3	1–5	3.02	0.88	−0.07	−0.49	0.60	0.61	0.39
Problems at home	3	1–5	1.84	0.86	1.13	0.90	0.65	0.65	0.49
Problems at work	3	1–5	2.00	0.90	0.81	0.01	0.60	0.63	0.45
Lack of necessities	4	1–5	1.57	0.59	1.61	3.32	0.67	0.68	0.51
Physical symptoms	5	1–5	2.13	0.46	1.23	1.85	0.69	0.69	0.53

*Skew, skewness; Kurt, kurtosis; α, Cronbach’s α coefficient of internal consistency; ω, McDonald’s omega total; AVE, average variance extracted*.

### Responses to Stress and the Perceived Impact of Stressors

Regarding the perceived impact of stressors during the COVID-19 epidemic, participants perceived the impact of confinement on their emotions as moderate, while they reported a low impact of problems at home and at work and no to a low impact of lack of necessities (Table 3). A minority of participants (25%) reported experiencing increased workload during the epidemic (Table 1).

On average, participants were characterized by moderate levels of neuroticism and low levels of vulnerability to stress (Table 3). The frequency distribution of ratings of their general health is shown in Table 1. A large majority reported good or excellent health.

Among other constructs, Table 3 also shows descriptive statistics for constructs related to emotional responses to stress. On average, participants reported experiencing low levels of negative emotions and infrequent to occasional perceived stress (loss of perceived control). They rarely felt anxious during the epidemic. Summing responses to GAD-7 items yielded an average total score of 4.28 points (*SD* = 3.81) on the 0–21 scale. A large percentage of participants scored 0 on GAD-7. For 76 (9%) of participants, the scale sum was above 10, which is considered the cutoff point for identifying moderate generalized anxiety. Thus, we conclude that our sample generally exhibited only mildly negative emotions at the time of our study. In terms of physical responses to stress, participants generally reported experiencing fatigue and sleepiness to a low degree, no (or only mild) physical pain and worsening of illness, and a slight increase in fatigue and decrease in sleep quality compared to pre-epidemic times (see Supplementary Table 1, part Physical Symptoms).

On average, participants reported mildly impaired cognitive function during the COVID-19 lockdown (Table 3). During the epidemic, they were slightly slower in performing their usual activities than before the epidemic, and they noticed slight negative changes in their speed of information processing, attention, memory, and executive control (Table 2). The mean CCQ score (4.18) was statistically significantly larger than 4 (the response indicating no change), *t*(829) = 8.90, *p* < 0.001, *d* = 0.31, 95% confidence interval for *d* = [0.17, 0.45], 1 − *β* = 1.00.

No statistically significant differences were found in subjective cognitive change by gender, age, education, relationship status, and employment status (Table 1).

### Predictors of Stress Responses and Subjective Cognitive Change

Supplementary Table 2 shows the correlations between different constructs used in the structural model for predicting physical symptoms and emotional responses to stress. Table 4 shows the standardized regression coefficients in the structural model. Among the COVID-19 crisis stressors, confinement showed the largest effect on all four predicted constructs (negative emotions, generalized anxiety, perceived stress, and physical symptoms). Perceived increased workload contributed to more intense physical symptoms. Problems at home and at work and a lack of necessities did not appear to contribute to the emotional and physiological responses to stress. Risk factors for stress showed an expected contribution to stress responses. Stress vulnerability, poor general health, female gender, and younger age contributed to all four types of stress responses, while neuroticism contributed only to emotional responses to stress but not to physical responses.

**Table 4:** Regression coefficients in the structural model for predicting the four mediator variables.

Predictor	Negative emotions		GAD-7		PSS-4		Physical symptoms	
	*b* (*SE*_b_)	*β*	*b* (*SE*_b_)	*β*	*b* (*SE*_b_)	*β*	*b* (*SE*_b_)	*β*
Confinement	1.49 (0.23)	0.51^***^	0.65 (0.11)	0.33^***^	0.35 (0.14)	0.18^*^	0.32 (0.10)	0.24^**^
Problems at home	0.03 (0.16)	0.01	−0.09 (0.11)	−0.05	0.10 (0.15)	0.05	0.04 (0.11)	0.03
Problems at work	−0.07 (0.12)	−0.02	−0.06 (0.10)	−0.03	0.06 (0.12)	0.03	0.12 (0.09)	0.09
Lack of necessities	−0.00 (0.11)	−0.00	0.05 (0.09)	0.03	0.17 (0.10)	0.09	0.15 (0.08)	0.11
Increased workload	0.13 (0.23)	0.02	0.22 (0.16)	0.05	−0.16 (0.17)	−0.04	0.55 (0.13)	0.18^***^
Vulnerability to stress	1.16 (0.17)	0.40^***^	0.97 (0.13)	0.49^***^	0.71 (0.13)	0.37^***^	0.31 (0.11)	0.23^***^
Neuroticism	0.33 (0.13)	0.11^*^	0.22 (0.10)	0.11^*^	0.53 (0.12)	0.27^***^	−0.01 (0.09)	−0.00
General health	−0.87 (0.16)	−0.22^***^	−0.76 (0.10)	−0.28^***^	−0.90 (0.11)	−0.34^***^	−0.63 (0.08)	−0.34^***^
Female gender	1.28 (0.26)	0.18^***^	1.00 (0.17)	0.21^***^	0.45 (0.18)	0.10^*^	0.33 (0.15)	0.10^*^
Age	−0.06 (0.01)	−0.31^***^	−0.04 (0.01)	−0.29^***^	−0.04 (0.01)	−0.32^***^	−0.01 (0.00)	−0.10^*^

* *p < 0.05*;

** *p < 0.01*;

*** *p < 0.001*.

In the model studied, the four types of stress reactions predicted subjective cognitive change and were able to explain 34% of variance in the CCQ score. The contributions of negative emotions (*b* = 0.14, *SE*_b_ = 0.05, *z* = 2.65, *p* = 0.004, *β* = 0.32) and physical symptoms (*b* = 0.40, *SE*_b_ = 0.05, *z* = 8.27, *p* < 0.001, *β* = 0.44) were statistically significant, whereas the contributions of generalized anxiety (*b* = −0.05, *SE*_b_ = 0.06, *z* = −0.78, *p* = 0.434, *β* = −0.07) and perceived stress in terms of loss of perceived control (*b* = −0.05, *SE*_b_ = 0.05, *z* = −0.98, *p* = 0.327, *β* = −0.07) did not reach statistical significance.

## Discussion

Our model of subjective cognitive change during the COVID-19 epidemic showed acceptable fit to the data collected. This suggests that the COVID-19 represented a stressful situation that elicited similar responses to those in other types of stressful situations. Confinement (including media coverage and worries about the latest news and other issues) and increased workload during the COVID-19 crisis – potentiated by vulnerability to stress, neuroticism, and poor general health – led to affective, physiological, and cognitive responses that resulted in subjective cognitive decline.

A very small, but statistically significant subjective cognitive decline was reported on average by our participants. Boals and Banks (2012) suggested that perceived stress during the COVID-19 crisis could lead to intrusive thoughts that compete for limited cognitive resources, cause mind wandering, and decrease academic, occupational, and daily life tasks performance. Among the most important factors for mind wandering, they cited worries about the latest news regarding the pandemic, worries about loved ones and others who might be at risk health-wise or financially, and worries about themselves. Overall, our results are consistent with their conjecture, but also reveal some further details.

### The Impact of Stressors on COVID-19 Stress Responses

Previous studies have shown that stressors have an important impact on negative emotions, depression, and anxiety (Stein and Lang, 2002; Kemeny, 2003; Scott et al., 2013; Chattarji et al., 2015; Zhaoyang et al., 2020). In our study, increased workload, most likely due to increased teleworking and the need to adapt to the new situation (adjusting daily schedule and work process to work from home, helping children with online learning, increased use of computers and digital communication, etc.) contributed to physical symptoms (i.e., increased fatigue, decreased sleep quality, physical pain, and exacerbation of illness). Among the specific stressors associated with COVID-19 crisis, confinement was found to be the single most important origin of affective responses to stress and physical symptoms. It is possible that this predictor covered other stressful aspects of COVID-19 lockdown, as it was positively associated with changes in work or study conditions, income reductions, and problems with relationships at home (see Supplementary Table 2).

Other studies also found confinement or its variants to be important stressors during the COVID-19 epidemic. For example, Xin et al. (2020) found that mandatory quarantine during the initial COVID-19 outbreak in China was associated with negative thoughts (perceived discrimination) and emotional distress. Tang et al. (2020) found that the likelihood of exhibiting generalized anxiety and depression was higher among respondents who were quarantined than those who were not. Ammar et al. (2021b) reported that COVID-19 home confinement negatively affects mental wellbeing and emotional status and leads to unhealthy diet behaviors. Trabelsi et al. (2021) confirmed the effects of confinement on impaired sleep quality and decreases in physical activity. Bai et al. (2004), who studied the effects of quarantine during the outbreak of various diseases, found an increase in exhaustion, anxiety, irritability, and insomnia. Similarly, our results show a very general effect of this stressor on emotional, cognitive, and physical functioning during the epidemic. The negative effect of confinement could be attributed to quarantine-induced boredom, frustrations, perceived loss of freedom, decreased physical activity, (daily) travel restrictions, altered schedule due to working or studying from home, altered sleep–wake rhythms, and anxiety due to myths, misinformation, erroneous news reports in the media, and misunderstanding of health-related messages (Bao et al., 2020; Saraswathi et al., 2020). The media could also contribute to the stigmatization of those infected and those who leave their homes (Gualano et al., 2020), contributing to distress. In addition, Gualano et al. (2020) found that internet use increased during the COVID-19 lockdown for three-quarters of participants, and using the internet as a source of information led to a higher likelihood of anxiety. In terms of the harmful effects of confinement, it is interesting to note that a higher prevalence of generalized anxiety disorder has also been found in prisoners (Costa et al., 2010; Dadi et al., 2016), where it has been attributed to increased exposure to deprivation of social interaction, deprivation of liberty, rigid rules, constant control of individuals and stressful situations, among other factors (Costa et al., 2010), and similar characteristics could be attributed to the COVID-19 lockdown.

### Other Factors of COVID-19 Stress Responses

In addition to the aforementioned effects of stressors, we observed an independent contribution of stress vulnerability, neuroticism, and poor general health to negative emotions, generalized anxiety, and perceived stress. Similar findings have been reported by other studies (Kemeny, 2003; Sexton et al., 2003; Schneider, 2004; Schneider et al., 2012; Scott et al., 2013; Warttig et al., 2013; Gallagher et al., 2014; Klainin-Yobas et al., 2014). Further, women reported more affective responses to stress and more physical symptoms. Gender differences in emotional, physiological, and cognitive responses to stress have also been observed in other studies (Warttig et al., 2013; Novais et al., 2017; Hodes and Epperson, 2019; Gualano et al., 2020; Liu et al., 2020). Several alternative explanations for these differences have been provided, ranging from neurobiological (Novais et al., 2017; Hodes and Epperson, 2019) to psychological in the sense that women are exposed to more stressors or perceive stressors as more stressful than men (Warttig et al., 2013). Finally, age acted as a preventive factor against affective responses to stress, as also found in several other studies (Warttig et al., 2013; Gualano et al., 2020). According to Warttig et al. (2013), older adults report fewer stressors than their younger counterparts because they are less active and redirect their preferences toward satisfying goals and emotion regulation to maximize positive emotional experiences and minimize negative ones. Also, physical symptoms may be perceived as normative in old age, so older people have a higher threshold for reporting them as potential stressors. In addition, the COVID-19 crisis likely brought fewer changes to the lives of older people (especially retirees) than to younger people.

More severe physical symptoms (i.e., greater increase in fatigue and worsening of sleep quality and health status) were reported by younger and more vulnerable individuals, by women, and by participants who reported increased workload, greater impact of confinement and poorer health. This is consistent with Wang et al.’s (2020) finding that anxiety was higher among students who reported poor health, and suggests that restricted movement during the epidemic may have exacerbated pre-existing health problems.

### The Association of Physical and Affective Stress Responses With Subjective Cognitive Decline

In our model, physical symptoms and affective responses to stress were considered mediator variables in the relationship between stressors and subjective cognitive change. The results are consistent with other studies that have found subjective cognitive complaints to be associated with physical health problems (Comijs et al., 2002; Jacob et al., 2019) and sleep problems (Stenfors et al., 2013; Miley-Akerstedt et al., 2018; Jacob et al., 2019); the variables included in the construct Physical Symptoms in our study. Comijs et al. (2002) speculated that physical problems may contribute to lower wellbeing and motivation, leading to poor performance on cognitive tasks and memory complaints.

Both subjective and objective cognitive decline have also previously been associated with negative emotions and anxiety (Comijs et al., 2002; Ouimet et al., 2009; Boals and Banks, 2012; Harlé et al., 2013; Okon-Singer et al., 2015; Hill et al., 2016; Jacob et al., 2019). In our study, we found no evidence of the association between subjective cognitive decline and generalized anxiety. One possibility for such a result could be that we found a prevalence of generalized anxiety disorder of 9% in our sample (this was the percentage of participants with GAD-7 sum greater than 10 points), which is much lower than what has been found in some other countries and subject groups, where typically about one-third of the samples had an anxiety disorder and about one-third to one-half had sleep disorders during the COVID-19 epidemic (Fu et al., 2020; Gualano et al., 2020; Huang and Zhao, 2020; Hyland et al., 2020; Twenge and Joiner, 2020; Fiorenzato et al., 2021). Unfortunately, there are no data for the prevalence of generalized anxiety disorder in Slovenia in normal times, but a 12-month prevalence of threshold GAD of about 2% was observed in the European community before the COVID-19 pandemic (Lieb et al., 2005) and a 1-month prevalence of about 8% was observed in primary care patients worldwide (Maier et al., 2000), suggesting that the prevalence of GAD was only slightly increased in our sample. The use of other, more discriminating measures of anxiety could lead to different results. The same could be true for the measure of perceived stress (PSS-4), which had low reliability. This could be one of the reasons why we found no association between the PSS-4 scale score and the CCQ score, which is not consistent with previous studies reporting that loss of perceived control is related to subjective cognitive complaints (Comijs et al., 2002; Boals and Banks, 2012; dos Santos et al., 2012; Steinberg et al., 2013).

### Study Limitations and Strengths

Our study has several limitations. We used only self-report scales, which may have led to social desirability and other response biases. Second, the study was cross-sectional. At the time of our study, the epidemic had been declared in Slovenia for just over a month. The differences between our study and others in the expression of affective responses to stress could be explained by the different timing during the lockdown and the different measurement instruments used. Third, participation was voluntary, and stressed individuals may have a greater need to participate in studies, such as ours to express their concerns and problems. Fourth, snowball sampling was used, resulting in an unbalanced gender and age structure of the sample (with males and individuals older than 75 years underrepresented), so our results may not be generalizable to the general population. Fifth, as is common with online surveys, a number of participants (27%) left the survey before completing it or did not answer all questions, so attrition bias may be present (see Supplementary Table 3 and accompanying text for more information on this). Sixth, other relevant stressors (e.g., reduction in physical activity and social interactions) and constructs (e.g., depression) could be included in the model and instruments with better psychometric properties could be used instead of single indicator variables, but this would increase the length of the already long survey and lead to additional dropouts. Seventh, our model is unidirectional and predicts subjective cognitive change based on physical symptoms and affective responses to stress. However, the relationship between the constructs under study may be bidirectional. For example, negative emotions may increase subjective cognitive decline, and subjective cognitive decline may increase negative emotions. The lack of temporal order in the measurement of stress and subjective cognitive decline prohibits causal inferences, and the mediations in our structural model should not be interpreted as causal mediations (Maxwell and Cole, 2007). Longitudinal observation would be desirable to shed more light on psychological responses to the COVID-19 epidemic. Qualitative studies should be conducted as a complement to quantitative studies to investigate in more detail how participants experienced the lockdown and in which situations subjective cognitive decline occurred, how it changed over time and why, how the intensive use of digital technology affected it, etc. Finally, using objective measures of stress (e.g., measuring cortisol levels) to monitor physiological changes during the epidemic would provide a deeper understanding of the impact of the epidemic on physical and mental health. Future studies should also use tests of attention, memory, and executive function and address the potential objective cognitive decline caused by chronic stress due to the COVID-19 epidemic, as subjective and objective cognitive decline do not necessarily overlap in healthy adults (Markova et al., 2017; Barbe et al., 2018).

Nevertheless, we can say that our research is important because we collected the data during the critical period of the first wave of the COVID-19 epidemic, i.e., under lockdown and particular psychological circumstances, and our sample was large. A majority of our results were consistent with findings in the literature, which gives us confidence. Additional support for our findings comes from a very recently published study by Fiorenzato et al. (2021). They investigated the effect of lockdown on the mental health and cognitive functioning of Italian residents. Some findings overlap with ours. For example, their participants reported increased distress and decreased sleep quality. They also complained about their attention, temporal orientation, and executive functions during lockdown. Subjective cognitive complaints were associated with home confinement. In their study, subjective cognitive complaints were also associated with increases in anxiety and depression, female gender, younger age, and underemployment. In our study, gender and age had similar effects on mediator variables (physical symptoms and affective stress responses) but not directly on subjective cognitive decline.

An important contribution of our study is also the developed CCQ, a brief measure of recent subjective cognitive changes. The instrument showed adequate psychometric properties and could be used and further validated in the future studies on the influence of crisis situations on subjective cognitive decline.

### Conclusion

Our study showed that prolonged confinement can cause distress and lead to generalized anxiety, negative emotions, loss of perceived control, increased physical symptoms, and subjective cognitive decline. These results have several practical implications. First, governments should ensure that the experience of confinement is as tolerable as possible by setting a reasonable duration of lockdown and providing basic supplies and services (Li et al., 2020). Regulations designed to prevent the further spread of the virus must be well thought out and properly communicated to mitigate stress and prevent stress reactions. Interventions delivered *via* the Internet, mobile devices, or other types of media should be offered during confinement to monitor physical, mental, and psychosocial health, promote healthy lifestyles, and provide psychosocial support, especially for vulnerable groups, such as the elderly (see Ammar et al., 2021a). Second, cognitive impairment during the pandemic is inevitable for even the most resilient individuals (Boals and Banks, 2020). Therefore, attention should be paid to subjective (and objective) cognitive decline and to adapting work or learning processes during the epidemic. Expectations of what is realistic in times of crisis should be scaled down. In relation to stress, cognitive activities may have a preventive effect, as it has been shown that higher cognitive load focuses attention more on the neutral or positive non-threatening stimuli and reduces mind wandering toward worry (Najmi et al., 2015). However, according to the results of our study, increased workload can increase physical symptoms, such as fatigue and sleep problems. Therefore, a carefully planned, balanced level of work or study activities would be preferable during these difficult times.

## Data Availability Statement

The raw data supporting the conclusions of this article will be made available by the authors, without undue reservation.

## Ethics Statement

The studies involving human participants were reviewed and approved by The Ethics Committee of the Faculty of Arts at University of Ljubljana. The patients/participants provided their written informed consent to participate in this study.

## Author Contributions

AP and VK were involved in developing the theory and survey, planning the study, and distributed the invitations. AP set up the online survey, analyzed the data with the help of LK, and wrote the manuscript with assistance from VK. AP, LK, and VK discussed the results. LK prepared Figure 1. LK and VK commented on the manuscript. All authors contributed to the article and approved the submitted version.

## Conflict of Interest

The authors declare that the research was conducted in the absence of any commercial or financial relationships that could be construed as a potential conflict of interest.

## Publisher’s Note

All claims expressed in this article are solely those of the authors and do not necessarily represent those of their affiliated organizations, or those of the publisher, the editors and the reviewers. Any product that may be evaluated in this article, or claim that may be made by its manufacturer, is not guaranteed or endorsed by the publisher.
