# Supplementary material for: The Relationship Between Perceived Stress and Subjective Cognitive Decline During the COVID-19 Epidemic
Source: Front Psychol. 2021 Aug 5;12:647971. doi: 10.3389/fpsyg.2021.647971 (PMC8374330; doi:10.3389/fpsyg.2021.647971)
Supplement: Supplementary file 1 [file Data_Sheet_1.docx]

Supplementary Material

**Supplementary Table 1**

*The measurement models for predictor and mediator constructs – Item content, descriptive statistics, and factor loadings (N = 830)*

| Construct | *M* | *SD* | Skew | Kurt | λ |
| --- | --- | --- | --- | --- | --- |
| GAD-7 (Generalized anxiety) |  |  |  |  |  |
| Feeling nervous, anxious or on edge | 0.70 | 0.72 | 0.95 | 0.96 | .88^***^ |
| Not being able to stop or control worrying | 0.53 | 0.68 | 1.13 | 0.91 | .84^***^ |
| Worrying too much about different things | 0.69 | 0.76 | 0.96 | 0.56 | .85^***^ |
| Trouble relaxing | 0.77 | 0.78 | 0.90 | 0.56 | .86^***^ |
| Being so restless that it is hard to sit still | 0.32 | 0.61 | 2.01 | 3.92 | .74^***^ |
| Becoming easily annoyed or irritable | 0.72 | 0.77 | 0.90 | 0.42 | .84^***^ |
| Feeling afraid as if something awful might happen | 0.55 | 0.67 | 1.16 | 1.45 | .65^***^ |
| Negative emotions |  |  |  |  |  |
| Anger directed toward others | 2.13 | 1.00 | 0.69 | –0.05 | .60^***^ |
| Anger directed toward oneself | 1.72 | 0.90 | 1.26 | 1.20 | .74^***^ |
| Sadness | 2.16 | 1.01 | 0.70 | –0.04 | .75^***^ |
| Fear | 2.28 | 0.97 | 0.52 | –0.16 | .72^***^ |
| Worry | 2.80 | 1.02 | 0.25 | –0.45 | .72^***^ |
| Annoyance | 2.45 | 1.06 | 0.35 | –0.51 | .81^***^ |
| Depression | 1.78 | 0.92 | 1.11 | 0.77 | .83^***^ |
| Distracted thinking | 1.92 | 1.01 | 0.88 | –0.04 | .70^***^ |
| Longing for normality | 3.33 | 1.15 | –0.26 | –0.78 | .61^***^ |
| Loneliness | 2.12 | 1.07 | 0.81 | 0.05 | .69^***^ |
| PSS-4 (Loss of perceived control) |  |  |  |  |  |
| Unable to control important things | 2.82 | 1.06 | 0.11 | –0.51 | .79^***^ |
| Confident in ability to handle problems^†^ | 3.84 | 0.90 | –0.92 | 1.15 | –.44^***^ |
| Difficulties piling up | 2.10 | 0.90 | 0.65 | 0.30 | .74^***^ |
| Things were going your way^†^ | 3.28 | 0.87 | –0.53 | 0.35 | –.52^***^ |
| Vulnerability to stress |  |  |  |  |  |
| A sense of imbalance | 2.05 | 1.03 | 0.62 | –0.60 | .74^***^ |
| Self-denial | 1.43 | 0.69 | 1.65 | 2.47 | .69^***^ |
| Lack of resilience | 1.80 | 0.93 | 1.16 | 0.97 | .84^***^ |
| Vulnerability | 2.05 | 0.99 | 0.67 | –0.26 | .79^***^ |
| Tendency to suppress emotions | 1.79 | 0.96 | 1.14 | 0.70 | .72^***^ |
| Lack of family support | 1.56 | 0.89 | 1.80 | 3.12 | .65^***^ |
| Lack of social support | 1.90 | 1.03 | 0.95 | 0.06 | .62^***^ |
| Perfectionistic tendencies | 1.97 | 1.14 | 0.97 | –0.10 | .60^***^ |
| Poor adaptability | 1.56 | 0.79 | 1.43 | 1.80 | .72^***^ |
| Lacking confidence | 1.73 | 0.96 | 1.33 | 1.22 | .80^***^ |
| Lacking ways to deal with problems | 1.72 | 0.91 | 1.32 | 1.50 | .78^***^ |

**Supplementary Table 1 – continued**

| Construct | *M* | *SD* | Skew | Kurt | λ |
| --- | --- | --- | --- | --- | --- |
| Neuroticism |  |  |  |  |  |
| Emotional stability (reverse coded) | 2.45 | 0.84 | 0.30 | 0.10 | .65^***^ |
| Being worry free (reverse coded) | 3.44 | 0.97 | –0.21 | –0.24 | .71^***^ |
| Confinement |  |  |  |  |  |
| Time spent indoors | 2.40 | 1.14 | 0.44 | –0.56 | .70^***^ |
| Media coverage | 3.21 | 1.14 | –0.17 | –0.70 | .57^***^ |
| Restricted freedom of movement | 3.43 | 1.25 | –0.29 | –0.93 | .61^***^ |
| Problems at home |  |  |  |  |  |
| Family relationships | 2.01 | 1.16 | 1.03 | 0.19 | .68^***^ |
| Intimate partner relationship | 1.74 | 1.11 | 1.54 | 1.54 | .63^***^ |
| Reduced privacy | 1.77 | 1.10 | 1.39 | 1.05 | .72^***^ |
| Problems at work |  |  |  |  |  |
| Work-related problems | 2.43 | 1.33 | 0.51 | –0.92 | .60^***^ |
| Academic problems | 1.75 | 1.16 | 1.40 | 0.81 | .72^***^ |
| Economic problems | 1.81 | 1.11 | 1.33 | 0.90 | .56^***^ |
| Lack of necessities |  |  |  |  |  |
| Lack of personal protective equipment | 2.09 | 1.09 | 0.83 | –0.07 | .67^***^ |
| Lack of food | 1.12 | 0.42 | 4.36 | 23.00 | .73^***^ |
| Difficulties in accessing a doctor | 1.79 | 1.08 | 1.35 | 0.97 | .62^***^ |
| Lack of medicines | 1.27 | 0.65 | 2.82 | 8.36 | .69^***^ |
| Physical symptoms |  |  |  |  |  |
| Change in fatigue | 3.06 | 0.90 | –0.27 | –0.20 | .67^***^ |
| Change in sleep quality | 3.08 | 0.71 | –0.10 | 0.79 | .61^***^ |
| Physical pain | 1.42 | 0.83 | 2.12 | 4.07 | .52^***^ |
| Fatigue/sleepiness | 1.96 | 1.04 | 0.89 | 0.02 | .78^***^ |
| Worsening of illness | 1.31 | 0.70 | 2.62 | 7.02 | .72^***^ |

*Note.* Kurt = kurtosis. λ = standardized factor loadings. On all items, responses covered the entire possible range.

^†^This item was scored reverse before calculating the scale score.

^***^*p* < .001.

**Supplementary Table 2**

*Correlations between the constructs (N = 830)*

| Variable | 1 | 2 | 3 | 4 | 5 | 6 | 7 | 8 | 9 | 10 | 11 | 12 | 13 | 14 | 15 |
| --- | --- | --- | --- | --- | --- | --- | --- | --- | --- | --- | --- | --- | --- | --- | --- |
| 1 – Confinement | 1.00 | .38 | .29 | .27 | .03 | .44 | .18 | –.05 | .16 | –.08 | .62 | .48 | .37 | .26 | .28 |
| 2 – Problems at home | .38 | 1.00 | .37 | .18 | .10 | .45 | .17 | –.07 | .05 | –.22 | .49 | .40 | .36 | .29 | .22 |
| 3 – Problems at work | .29 | .37 | 1.00 | .24 | .23 | .33 | .15 | .05 | .13 | –.33 | .36 | .30 | .27 | .28 | .14 |
| 4 – Lack of necessities | .27 | .18 | .24 | 1.00 | .03 | .25 | .11 | –.29 | .07 | .10 | .30 | .27 | .24 | .35 | .14 |
| 5 – Increased workload | .03 | .10 | .23 | .03 | 1.00 | .06 | .02 | .07 | .04 | –.20 | .07 | .08 | .00 | .14 | .05 |
| 6 – Vulnerability to stress | .44 | .45 | .33 | .25 | .06 | 1.00 | .48 | –.16 | .20 | –.23 | .75 | .72 | .60 | .40 | .28 |
| 7 – Neuroticism | .18 | .17 | .15 | .11 | .02 | .48 | 1.00 | –.22 | .16 | –.18 | .43 | .43 | .44 | .21 | .12 |
| 8 – General health | –.06 | –.08 | .07 | –.24 | .06 | –.17 | –.21 | 1.00 | .03 | –.30 | –.12 | –.15 | –.20 | –.22 | –.15 |
| 9 – Gender (female) | .16 | .05 | .13 | .07 | .04 | .20 | .16 | .04 | 1.00 | –.16 | .20 | .19 | .09 | .10 | .04 |
| 10 – Age | –.07 | –.22 | –.37 | .11 | –.19 | –.24 | –.18 | –.30 | –.16 | 1.00 | –.27 | –.25 | –.17 | –.15 | –.01 |
| 11 – Negative emotions | .62 | .49 | .36 | .30 | .07 | .75 | .43 | –.12 | .20 | –.27 | 1.00 | .78 | .60 | .45 | .37 |
| 12 – GAD-7 | .48 | .40 | .30 | .27 | .08 | .72 | .43 | –.17 | .19 | –.21 | .78 | 1.00 | .57 | .40 | .31 |
| 13 – PSS-4 | .37 | .36 | .27 | .24 | .00 | .60 | .44 | –.20 | .09 | –.18 | .60 | .57 | 1.00 | .29 | .25 |
| 14 – Physical symptoms | .26 | .29 | .28 | .35 | .14 | .40 | .21 | –.26 | .10 | –.14 | .45 | .40 | .29 | 1.00 | .28 |
| 15 – CCQ | .28 | .22 | .14 | .14 | .05 | .28 | .12 | –.11 | .04 | .01 | .37 | .31 | .25 | .28 | 1.00 |

*Note.* Spearman coefficients were calculated to describe the correlations of general health and age with other variables; Pearson correlation coefficients were used in other cases. Correlations above .07 were statistically significant at *p* < .05, those above .09 were significant at *p* < .01, and those above .11 were significant at *p* < .001.

**Supplementary Table 3**

*Comparison of the participants who completed the survey (n = 830) with those who completed it only partially*

|  | Total completion | |  | Partial completion | | |  | Welch *t*-test | | |
| --- | --- | --- | --- | --- | --- | --- | --- | --- | --- | --- |
| Variable | *M* | *SD* |  | *n* | *M* | *SD* |  | *t* | *df* | *p* |
| Problems at work | 2.00 | 0.90 |  | 190 | 1.96 | 0.83 |  | –0.57 | 297.8 | .572 |
| Confinement | 3.02 | 0.88 |  | 174 | 3.00 | 0.98 |  | –0.24 | 265.7 | .808 |
| Lack of necessities | 1.57 | 0.59 |  | 174 | 1.60 | 0.62 |  | 0.60 | 241.3 | .549 |
| Problems at home | 1.84 | 0.86 |  | 169 | 1.77 | 0.77 |  | –0.13 | 233.8 | .896 |
| Negative emotions | 2.27 | 0.70 |  | 164 | 2.40 | 0.82 |  | 0.74 | 211.1 | .462 |
| GAD-7 | 0.61 | 0.54 |  | 144 | 0.72 | 0.66 |  | 1.84 | 178.7 | .068 |
| Vulnerability to stress | 1.78 | 0.66 |  | 102 | 1.99 | 0.75 |  | 1.25 | 121.4 | .213 |
| Neuroticism | 2.94 | 0.77 |  | 95 | 2.98 | 0.79 |  | –0.40 | 115.6 | .688 |
| General health | 3.07 | 0.73 |  | 95 | 2.84 | 0.75 |  | –2.88 | 115.4 | .005 |
| PSS-4 | 2.45 | 0.66 |  | 92 | 2.66 | 0.62 |  | 3.04 | 115.7 | .003 |
| CCQ | 4.18 | 0.59 |  | 56 | 4.14 | 0.53 |  | –0.54 | 64.5 | .594 |
| Physical symptoms | 2.13 | 0.46 |  | 55 | 2.08 | 0.45 |  | –0.85 | 61.7 | .397 |
| Age | 45.82 | 16.28 |  | 61 | 55.17 | 16.78 |  | 4.21 | 68.6 | < .001 |
|  | Total completion | |  | Partial completion | | |  | Chi-square test | | |
|  | *f* | % |  |  | *f* | % |  | χ^2^ | *df* | *p* |
| Gender |  |  |  |  |  |  |  | 0.40 | 1 | .528 |
| Male | 183 | 22 |  |  | 10 | 18 |  |  |  |  |
| Female | 647 | 78 |  |  | 47 | 82 |  |  |  |  |
| Increased workload |  |  |  |  |  |  |  | 5.39 | 1 | .020 |
| Yes | 623 | 75 |  |  | 17 | 55 |  |  |  |  |
| No | 207 | 25 |  |  | 14 | 45 |  |  |  |  |

A note on possible attrition bias: Compared with those who completed the survey and answered all items included in our study, participants who exited the survey after reporting general health (*n* = 95) had slightly poorer general health, and participants who exited the survey after reporting data on the PSS-4 (*n* = 92) had slightly higher PSS-4 scores. This suggests that the negative impact of the COVID-19 epidemic may be slightly underestimated in our study. Participants who dropped out after being asked their age (*n* = 61) were slightly older than those who completed the survey. Among those who dropped out after being asked about workload (*n* = 31), a lower percentage reported increased workload. However, because information on a particular variable could not be obtained for participants who exited the survey before providing data for that variable, it is difficult to draw firm conclusions about attrition bias.
